# Propofol‐Facilitated Recovery Sleep Modulates Cognitive Function via Slow Oscillation‐Spindle Coupling After Acute Sleep Deprivation in Rats

**DOI:** 10.1002/cns.71076

**Published:** 2026-08-20

**Authors:** Chenyi Yang, Huan Liu, Xinyi Wang, Lin Zhang, Haiyun Wang

**Affiliations:** ^1^ Nankai University Tianjin China; ^2^ Department of Anesthesiology Central Hospital, Tianjin University (Formerly Tianjin Third Central Hospital) Tianjin China; ^3^ Tianjin University Tianjin China; ^4^ Tianjin Key Laboratory of Extracorporeal Life Support for Critical Diseases Tianjin China; ^5^ The Third Central Clinical College of Tianjin Medical University Tianjin China

**Keywords:** cognitive function, propofol, sleep, sleep deprivation, slow oscillations, spindles

## Abstract

**Background:**

Sleep deprivation (SD) is common but underrecognized in intensive care unit (ICU) patients and contributes to delirium and long‐term neurocognitive deficits. Propofol is widely used for titratable ICU sedation and has potential neuroprotective effects, but the mechanisms underlying its cognitive benefits during recovery sleep remain unclear. Evidence shows that coupling between slow oscillations (SO) and spindles during sleep is associated with memory consolidation processes. We hypothesized that propofol‐facilitated recovery sleep after acute SD would strengthen SO–spindle coupling aligned to the SO up state and thereby improve cognition.

**Methods:**

Male Sprague–Dawley rats (8–12 weeks, total *n* = 67) were assigned to control (CON), SD without intervention (SDN), spontaneous recovery (SDS), or propofol‐supported recovery (SDP). All SD groups underwent 48 h of total deprivation. During recovery, SDP received propofol (20 mg·kg^−1^·h^−1^, 6 h), whereas SDS recovered naturally. Spatial learning and memory were tested by the Barnes maze (BM); recognition memory by 2‐h and 24‐h novel object recognition (NOR) for short‐ and long‐term recognition memory. Sleep electroencephalography (EEG) and electromyography (EMG) were recorded for 12 h during recovery in SDS and SDP. We quantified SO and spindle parameters and SO–spindle phase coupling. Separate animals were analyzed for cortical Na^+^‐K^+^‐2Cl^−^ cotransporter 1 (NKCC1)/K^+^‐Cl^−^ cotransporter 2 (KCC2) expression ratio.

**Results:**

Sleep deprivation impaired BM and NOR performance. Propofol during recovery restored spatial performance and 24‐h recognition retention. Propofol also increased SO density compared with SDS (11.13 ± 0.14 vs. 9.49 ± 1.26 events/min, *p* = 0.040) and improved phase‐accurate SO–spindle alignment, with higher phase‐vector projection (Vproj; 0.30 ± 0.11 vs. −0.33 ± 0.37, *p* = 0.036), increased up‐phase events (0.38 ± 0.01 vs. 0.16 ± 0.10, *p* = 0.036), and significant up‐state preference only in SDP (*V*‐test, *p* = 0.035). Molecularly, propofol decreased the NKCC1/KCC2 ratio by reducing NKCC1 and increasing KCC2.

**Conclusion:**

Propofol‐facilitated recovery sleep supports spatial and recognition memory, accompanied by up‐state‐precise SO–spindle coupling and reduced NKCC1/KCC2. These findings suggest SO–spindle alignment may serve as a candidate EEG marker of recovery sleep and highlight chloride‐transport‐related pathways as therapeutic targets for SD‐related cognitive impairment.

## Graphical abstract

This study examined how propofol‐supported recovery sleep shapes cognitive and sleep‐EEG outcomes after 48‐h SD. Rats received Barnes maze, 2‐h and 24‐h NOR testing, cortical EEG/EMG recording, and NKCC1/KCC2 expression analysis. Propofol increased slow‐oscillation density and restored precise SO–spindle alignment, accompanied by a shift in NKCC1/KCC2 expression, indicating a sleep‐EEG phenotype linked to cognitive recovery.

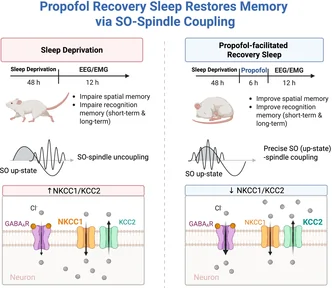

## 1 Introduction

Sleep deprivation (SD) is a common and underrecognized complication in intensive care unit (ICU) patients [1, 2]. Recent reports showed 66% poor sleep quality or severe disturbance during ICU stay [3] and up to 74% in nonsedated cohorts [4]. This disruption includes markedly reduced total sleep time, fragmentation of sleep architecture, and loss of restorative deep sleep stages on polysomnography [5]. In the ICU, acute SD has been linked to impaired cognition, emotional dysregulation, and delirium [6, 7] and is associated with worse long‐term neurocognitive outcomes when delirium occurs [8, 9]. Even after discharge, many ICU survivors report persistent sleep disturbances, more than 50% at 1 month and remaining elevated to 12 months or longer, and ongoing cognitive impairments [6, 10], underscoring the need for strategies that promote effective recovery sleep and neuroprotection during and after critical illness.

In the ICU, propofol is widely used for titratable sedation of mechanically ventilated patients and is recommended over benzodiazepines in contemporary practice guidelines [5]. Beyond sedation, converging evidence indicates neuroprotective actions relevant to cognition, including attenuation of glutamate‐mediated excitotoxicity, potentiation of GABAergic inhibition, suppression of microglial‐driven neuroinflammation, and reduction of oxidative stress [11]. Electrophysiologically, propofol engenders prominent slow–delta and coherent frontal alpha oscillations that resemble dynamics of natural non‐rapid eye movement (NREM) sleep [12, 13, 14]. Along this line, our prior work in 48‐h sleep‐deprived rats showed that propofol restored disturbed sleep architecture and enhanced NREM *δ* power [15]. However, how propofol‐associated recovery sleep alters specific sleep‐EEG features linked to cognitive outcomes remains unclear.

During NREM sleep, cortical slow oscillations (SOs; < 1 Hz up/down‐state transitions) organize alternating windows of cortical depolarization and hyperpolarization that gate thalamocortical interactions [16]. In humans, SOs often manifest as traveling waves across cortex, providing a macroscopic temporal scaffold [17]. Within this cycle, sleep spindles cluster at specific SO phases, indicating phase‐locked grouping rather than random co‐occurrence [18]. The precision of SO–spindle timing, rather than event counts alone, has been associated with memory outcomes in humans, and age‐related decoupling is associated with overnight forgetting [19]. Across younger and older adults, more precise up‐state coupling supports consolidation [20]. Recent evidence further extends this relationship beyond classic declarative memory paradigms, showing that stronger SO–spindle coupling also predicts sequence‐based language learning [21]. Importantly, human work also suggests that weakly encoded memories after acute sleep restriction can be rescued following one night of recovery sleep, and that SO–spindle coupling during the recovery night is associated with this mnemonic benefit, particularly for vulnerable memory traces [22]. Consistent with these observations, rodent studies show post‐learning adjustments in spindle timing relative to slow activity that track offline performance gains [23]. Together, these findings suggest that SO–spindle coupling during recovery sleep may provide an informative electrophysiological readout for evaluating whether restoration of sleep‐dependent temporal coordination is associated with cognitive recovery after prior sleep loss.

In this study, we hypothesized that propofol‐facilitated recovery sleep promotes memory consolidation after SD, linked to more precise alignment of spindles to the SO up‐state. To evaluate spatial learning and recognition memory, rats in the control, SD without intervention, spontaneous recovery, and propofol‐supported recovery groups were tested using the Barnes maze (BM) and 2‐h and 24‐h novel object recognition (NOR) tests, reflecting short‐term and longer‐term recognition memory, respectively. To interrogate SO–spindle coupling, cortical EEG and EMG were recorded; we quantified SO and spindle density, amplitude, frequency, and duration, and assessed phase accuracy of SO–spindle coupling across groups. In parallel, cortical NKCC1/KCC2 expression was measured to explore a candidate molecular mechanism for coupling precision. Together, this study aimed to delineate propofol‐modulated sleep EEG phenotypes relevant to memory consolidation after SD, and to suggest tractable intervention targets for deprivation‐induced memory impairment.

## 2 Methods

### 2.1 Animals

Male Sprague–Dawley rats (280–350 g) were obtained from Beijing Vital River Laboratory Animal Technology Co. Ltd. Animals were housed individually with ad libitum access to food and water under a 12 h light/dark cycle (lights on 07:00–19:00) at 21°C–24°C. Animals were randomly allocated to four groups (CON, SDS, SDP, and SDN); details of group procedures and testing windows are described in Experimental design and timeline. Behavioral scoring and data analysis were performed by investigators who were unaware of group allocation.

A total of 67 rats were used in this study across three independent cohorts. The behavioral cohort included 10 rats per group (CON, SDN, SDS, and SDP) for BM and NOR testing. A separate EEG/EMG cohort was used for recovery‐period electrophysiological analysis (CON, *n* = 3; SDS, *n* = 5; SDP, *n* = 3). An additional western blot cohort included four rats per group for molecular analysis. Separate cohorts were used to preserve the temporal specificity of post‐deprivation assessments and to minimize potential interference of behavioral testing, particularly the light and sound exposure associated with the BM, with the effects of SD and recovery intervention.

### 2.2 Experimental Design and Timeline

**FIGURE 1:**
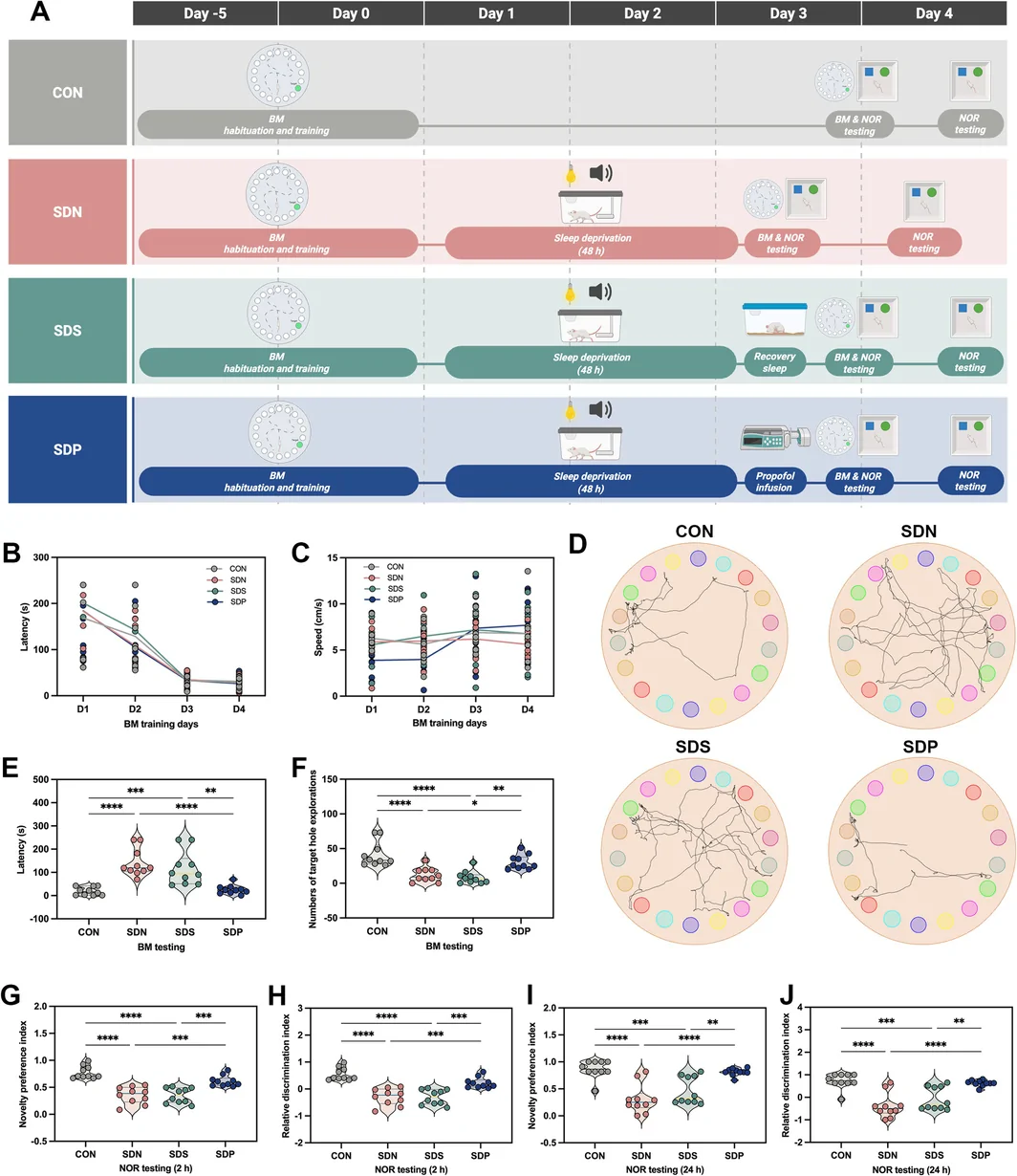
Behavioral effects of sleep deprivation and recovery. (A) Timeline of the behavioral cohort for the four groups. SDN: 48 h sleep deprivation from Day 1 at 07:00 to Day 3 at 07:00, followed immediately by BM probe testing and NOR with a 2‐h retention interval on Day 3; the 24‐h NOR was performed on Day 4. SDS: 48 h SD followed by natural recovery sleep on Day 3, with behavioral testing performed immediately thereafter; the 24‐h NOR was performed on Day 4. SDP: 48 h SD followed by a 6‐h propofol infusion during the recovery period on Day 3, with behavioral testing performed immediately thereafter; the 24‐h NOR was performed on Day 4. CON: No sleep manipulation and behavioral testing at matched time points. BM habituation and training were performed from Days −5 to −1, and Day 0 was reserved for consolidation. (B, C) BM training across 4 days: (B) escape latency and (C) path speed. (D) Representative BM probe trajectories for each group. (E, F) BM probe outcomes on the test day: (E) escape latency to the target hole and (F) number of target‐hole crossings. (G, H) NOR performance with a 2‐h retention interval: (G) novelty preference index and (H) discrimination index. (I, J) NOR performance with a 24‐h retention interval (same metrics as in G, H): (I) novelty preference index and (J) discrimination index. Data are shown as mean ± SD with individual animals overlaid; *n* = 10 rats per group. Statistics: Two‐way repeated‐measures ANOVA for BM training (group × day); one‐way ANOVA for BM probe and NOR outcomes across groups with Tukey post hoc. The *p* values refer to the differences of variables between the groups, *p < 0.05, **p < 0.01, ***p < 0.001, ****p < 0.0001.

The behavioral schedule is summarized in Figure 1A. Rats underwent BM habituation on Day −5 and BM training on Days −4 to −1. Day 0 was reserved for consolidation. SDN, SDS, and SDP received 48 h of SD from Day 1 at 07:00 to Day 3 at 07:00, whereas CON had no deprivation. SDN was tested immediately after SD with the BM probe, followed by NOR with a 2 h retention interval; the 24‐h NOR was performed on the following day. After SD, SDS underwent natural recovery sleep and was tested immediately thereafter with the same sequence; the 24‐h NOR was performed on the following day. SDP received propofol at 20 mg·kg^−1^·h^−1^ for 6 h during the recovery period after SD and was tested immediately thereafter with the same sequence; the 24‐h NOR was performed on the following day. CON followed a time‐matched behavioral timetable without sleep manipulation.

**FIGURE 2:**
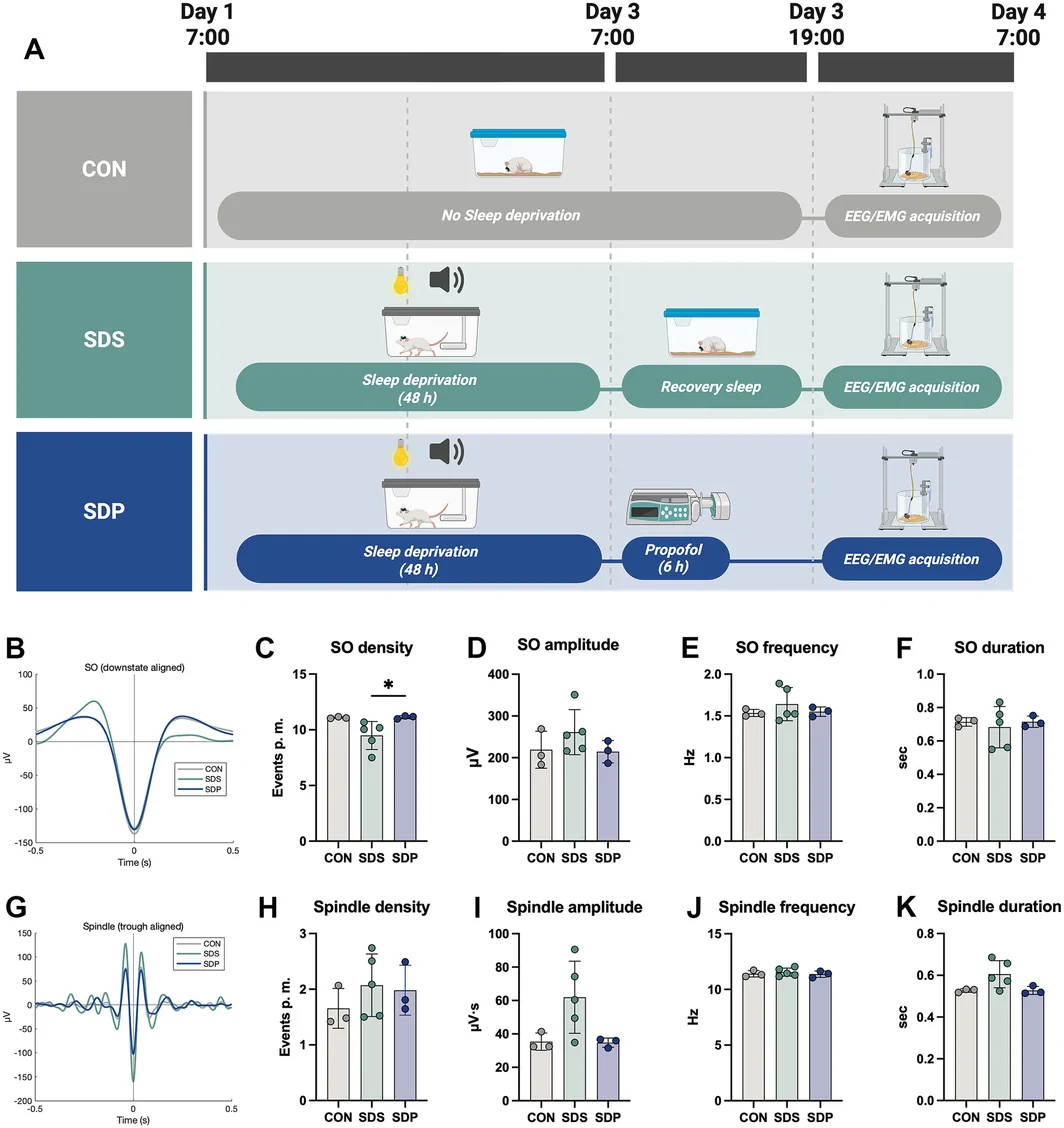
Recovery sleep SOs and spindles during SWS. (A) Recording windows for groups. (B) Grand‐average SO waveform. (C–F) SO metrics: (C) density differs across groups, whereas (D) amplitude, (E) frequency, and (F) duration are largely comparable. (G) Grand‐average spindle waveform. (H–K) Spindle metrics: (H) density, (I) amplitude, (J) frequency, and (K) duration were not significantly different across groups. Data are shown as mean ± SD with individual animals overlaid; *n* = 3–5 rats per group. Group comparisons were performed using Kruskal–Wallis tests, followed by Dunn's multiple‐comparisons test where appropriate. The *p* values refer to the differences of variables between the groups, *p < 0.05.

For sleep EEG/EMG, a separate rat cohort was used. CON, SDS, and SDP were recorded from Day 3 at 19:00 for 12 h, as summarized in Figure 2A. SDN did not undergo recovery sleep and therefore was not included in the EEG/EMG cohort.

A separate rat cohort was used to examine molecular substrates, as shown in Figure 4A. Cortical tissue was collected at matched time points: immediately after SD in SDN, after natural recovery in SDS, and after propofol‐supported recovery in SDP, with time‐matched CON collected in parallel for Western blot analyses.

### 2.3 Sleep Deprivation

SD was induced with an automated intermittent tactile‐stimulation system (Model 80391; Lafayette Instrument, Lafayette, IN, USA), as described in our previous studies [15, 24]. Rats were housed individually in the apparatus and exposed to periodic movement of a near‐silent metal bar that briefly contacted the animal to prevent consolidated sleep while allowing free ambulation and ad libitum access to food and water. The procedure was fully automated and avoided human handling or insertion of foreign objects, thereby minimizing handling‐related stress and experimenter bias. To approximate ICU‐like sleep disruption, the system operated in a controlled environment with elevated background noise and extended light exposure. SD was delivered continuously for 48 h, starting at 07:00.

### 2.4 Barnes Maze

Procedures followed our previous study [25]. Briefly, a circular platform (diameter 1215 mm) with 18 evenly spaced holes (diameter 100 mm) was used; only one hole served as the target, connected to a black escape box (170 × 120 × 60 mm). On Day 5, each rat was guided to the target hole and allowed to remain in the escape box for 4 min to habituate. From Days −4 to −1, rats received two training trials per day for four consecutive days; at the start of each trial, an opaque cylinder (20 cm diameter × 27 cm height) was placed at the center to restrain the animal for 10 s before release, and continuous white noise (75.8 dB) together with bright illumination (1000 lx) were provided as mild aversive cues to motivate escape. Beginning with the second training session, the maze platform was randomly rotated before each session while the escape box remained fixed in the same room orientation to discourage reliance on odor or intramaze cues; the platform and escape box were wiped with 75% ethanol between sessions. On the probe day, the escape box was removed, and rats were allowed to freely explore; primary latency was defined as the time to first locate the former target hole and investigate it for > 5 s.

### 2.5 Novel Object Recognition Test

NOR was conducted in a 40 × 40 × 40 cm arena with black walls, as described in our previous work [25]. On the NOR test day (Day 3 for SDN; Day 4 for SDS, SDP, and CON), rats first completed the scheduled BM testing and then underwent a brief arena habituation (5 min) immediately before NOR. The sample session consisted of two identical objects (A1 and A2) placed on the floor; each rat was introduced with its back facing the objects and allowed to explore for 10 min. After a 2 h retention interval in the home cage, the test session was performed by replacing one familiar object with a novel object (C), while the other remained as the familiar object (A3). Object identity and left/right positions were counterbalanced across animals and groups. The discrimination index (DI) was calculated as (T_C − T_A3)/(T_C + T_A3), where T_C and T_A3 denote the exploration time of the novel and familiar objects during the test session, respectively. The arena and objects were wiped with 75% ethanol between sessions to eliminate odor cues.

### 2.6 EEG/EMG Acquisition

Rats were anesthetized with isoflurane, and epidural stainless‐steel screw electrodes were implanted for EEG recording over the frontal cortex (AP +2.5 mm, ML ±1.5 mm) and the parietal cortex (AP −2.0 mm, ML ±1.5 mm); a ground/reference screw was placed over the cerebellum, as described in our previous study [15]. Flexible wire electrodes were inserted into the nuchal muscles for EMG. All surgeries were performed aseptically with perioperative monitoring and analgesia per institutional guidelines. After surgery, animals were singly housed for recovery and acclimation; implantation was completed at least 7 days before behavioral habituation, with tether/cable acclimation during the last 3 days. EEG/EMG leads were connected to a commutator and recorded with the Medusa system (Bio‐Signal Technologies, McKinney, TX, USA). In SDS and SDP groups, recovery‐period EEG/EMG was obtained on Day 3 (recording window approximately 19:00–07:00), following natural recovery sleep (SDS) or a 6 h propofol infusion (SDP) earlier that day; no EEG/EMG was collected in SDN since there was no recovery sleep. Signals were sampled at 1000 Hz and filtered offline using zero‐phase 4th‐order Butterworth filters: EEG 0.3–100 Hz with a 50 Hz notch; EMG 10–200 Hz.

### 2.7 EEG/EMG Analysis

#### 2.7.1 Sleep Staging

Sleep–wake stages were first auto‐scored with AccuSleep on 10‐s epochs using EEG and EMG, followed by manual review by three experienced scorers blinded to group; disagreements were resolved by consensus [26]. Final stages were Wake, NREM, and REM. For downstream analyses, NREM was further subdivided into SWS and pre‐REM according to established rodent criteria: Wakefulness was identified by mixed‐frequency EEG with sustained EMG activity; SWS by high‐amplitude low‐frequency activity (delta < 4 Hz) with reduced EMG tone; REM by low‐amplitude EEG dominated by theta activity (5–10 Hz) with phasic twitches and decreased EMG tone; pre‐REM (intermediate stage) by decreased delta, progressive theta increase, and the presence of sleep spindles [27, 28].

#### 2.7.2 SWS Oscillatory Events (SO and Spindle)

Before event detection, artifact‐contaminated periods (e.g., amplifier saturation, gross movement) were excluded, and event detection was parameterized according to previous studies [28]. SOs were identified after 0.3–4.5 Hz filtering using a zero‐crossing procedure with morphology constraints: two consecutive negative‐to‐positive zero crossings 0.4–2.0 s apart; within each rat/channel, candidates were restricted to the top 35% by negative‐peak amplitude and, from those, the top 45% by negative‐to‐positive peak‐to‐peak amplitude [29, 30, 31]. Spindles were detected after 7–20 Hz filtering by extracting the Hilbert envelope, smoothing with a 200‐ms moving average, and requiring envelope amplitude > 1.5 SD (relative to SWS in that channel) for 0.4–2.0 s (onset at first threshold crossing); spindle amplitude was quantified as the area under the envelope between onset and end [32].

#### 2.7.3 SWS SO–Spindle Coupling

Coupling was quantified by (i) co‐occurrence, defined as the spindle envelope maximum falling between SO onset and offset (SO boundaries from zero crossings), and (ii) phase coupling. For phase coupling, the raw EEG was filtered at 0.6–1.8 Hz to capture the asymmetric SO shape; the Hilbert transform provided the instantaneous SO phase, from which we sampled the SO phase at spindle maximum. We then computed: the preferred phase (circular mean), the mean vector length (MVL) as coupling strength, the Vproj onto the SO up‐state unit vector at 0° (positive values indicate an up‐state bias; negative values indicate displacement away from the up‐state toward down‐state phases), the fraction of up‐phase events defined as the proportion of spindle maxima within ±45° around 0°, and the pairwise phase consistency (PPC) as a bias‐free measure of across‐event phase agreement. Circular statistics and *V*‐tests for alignment toward the hypothesized SO up‐state (0°) were computed in MATLAB R2022b (MathWorks, Natick, MA, USA) [28, 29].

### 2.8 Sample Collection

Rats were terminally anesthetized and transcardially perfused with ice‐cold heparinized PBS (10 U/mL) until the effluent ran clear. The frontal and parietal cortical tissue was then rapidly dissected, snap‐frozen in liquid nitrogen, and stored at −80°C for Western blot analyses.

### 2.9 Western Blot

Tissue membrane proteins were extracted on ice using a membrane protein extraction kit (Thermo Fisher Scientific, Waltham, MA, USA; Cat. No. 89842) containing protease and phosphatase inhibitors. Protein concentration was determined by BCA assay. Equal protein amounts (30 μg) were separated by 10% SDS‐PAGE and transferred to 0.45 μm PVDF membranes. NKCC1 and KCC2 were run on separate gels and processed on separate membranes, each incubated overnight at 4°C with the corresponding rabbit monoclonal primary antibody (anti‐NKCC1, Abcam, Cambridge, UK; ab303518, 1:1000; anti‐KCC2, Abcam, Cambridge, UK; ab259969, 1:1000). A HRP‐conjugated goat anti‐rabbit secondary antibody (Abcam, Cambridge, UK; ab97051, 1:5000) was used for 1 h at room temperature. Signals were developed with ECL and quantified in ImageJ (NIH, Bethesda, MD, USA). Na^+^/K^+^‐ATPase (mouse monoclonal, Abcam, Cambridge, UK; ab7671, 1:5000) served as the loading control for normalization. To minimize KCC2 aggregation, samples were heated at 70°C for 10 min in the sample buffer rather than boiled.

### 2.10 Statistical Analyses

Statistical analyses for behavioral tests (BM, NOR) and Western blots were performed in GraphPad Prism 10 (GraphPad Software, San Diego, CA, USA). Data are reported as mean ± SD. Each rat was treated as an independent experimental unit for statistical analysis. All tests were two‐tailed, with *α* = 0.05 as the significance threshold.

Normality was assessed using the Shapiro–Wilk test, and homogeneity of variance was checked using the Brown–Forsythe test before parametric group comparisons. A Shapiro–Wilk *p* < 0.05 in one or more groups or a Brown–Forsythe *p* < 0.05 was considered a potential diagnostic concern for parametric analysis. Because normality tests have limited interpretability in small samples, diagnostic tests were treated as supportive rather than definitive. Non‐parametric sensitivity analyses for the principal outcomes were performed using Kruskal–Wallis tests followed by Dunn's multiple‐comparisons test where appropriate. Full diagnostic results and sensitivity analyses are provided in Table S1.

BM training (Days −4 to −1) was analyzed with a two‐way repeated‐measures ANOVA with group (CON, SDN, SDS, and SDP) and day (four training days); BM probe outcomes and NOR indices on the test day were analyzed with one‐way ANOVA across groups. Western blots (NKCC1, KCC2) were normalized to Na^+^/K^+^‐ATPase and compared by one‐way ANOVA; where applicable, the NKCC1/KCC2 ratio was analyzed likewise.

Following Fechner et al., metrics of SOs and spindles during SWS (e.g., event density, duration, amplitude, power) were quantified using the same event‐detection framework. For statistical analysis in the present study, values from the frontal and parietal channels were averaged for each rat, and group comparisons were then performed using Kruskal–Wallis tests, followed by Dunn's multiple‐comparisons test where appropriate. Co‐occurrence rates were analyzed after logit or arcsine‐square‐root transformation [28]. For SO–spindle phase coupling, circular statistics were computed in MATLAB R2022b (MathWorks, Natick, MA, USA) using the CircStat toolbox. *V*‐tests were used to assess whether spindle phases were significantly aligned to the hypothesized SO up‐phase within each group. Group differences in Vproj, the proportion of up‐phase spindles, mean vector length, and pairwise phase consistency were evaluated using Kruskal–Wallis tests, followed by Dunn's multiple‐comparisons test where appropriate.

## 3 Results

### 3.1 Propofol Restores Spatial and Recognition Memory After Sleep Deprivation

As shown in Figure 1A, rats were assigned to CON, SDN, SDS, or SDP. The BM and NOR tests were performed after the interventions. During BM acquisition, two‐way repeated‐measures ANOVA showed a significant day effect on escape latency (F(3, 144) = 92.35, *p* < 0.0001), but no significant group effect (F(3, 144) = 0.7694, *p* = 0.5129) or group × day interaction (F(9, 144) = 0.4171, *p* = 0.9244; Figure 1B). For path speed, there was also a significant day effect (F(3, 144) = 4.262, *p* = 0.0064), with no significant group effect (F(3, 144) = 0.9673, *p* = 0.4100) or group × day interaction (F(9, 144) = 1.567, *p* = 0.1306; Figure 1C). At the probe trial, one‐way ANOVA showed a significant group effect on escape latency (F (3, 36) = 16.27, *p* < 0.0001; Figure 1E). SDN significantly increased escape latency compared with CON (141.10 ± 59.26 vs. 19.16 ± 17.55 s, *p* < 0.0001; Figure 1E). Propofol treatment during recovery significantly shortened escape latency relative to both SDN and SDS (25.75 ± 19.29 vs. 141.10 ± 59.26 s, *p* < 0.0001; 25.75 ± 19.29 vs. 115.70 ± 72.74 s, *p* = 0.001; Figure 1E), with no significant difference from CON (*p* = 0.990; Figure 1E). One‐way ANOVA also showed a significant group effect on target‐hole crossings (F (3, 36) = 15.44, *p* < 0.0001; Figure 1F). SDN markedly reduced target‐hole crossings compared with CON (12.00 ± 10.27 vs. 41.30 ± 18.00, *p* < 0.0001; Figure 1F). Propofol treatment increased target‐hole crossings relative to both SDN and SDS (30.30 ± 10.54 vs. 12.00 ± 10.27, *p* = 0.012; 30.30 ± 10.54 vs. 8.30 ± 9.25, *p* = 0.002; Figure 1F), again with no significant difference from CON (*p* = 0.220; Figure 1F).

In the 2‐h NOR test, one‐way ANOVA revealed significant group effects on both the novelty preference index (F (3, 36) = 25.92, *p* < 0.0001; Figure 1G) and discrimination index (F (3, 36) = 25.92, *p* < 0.0001; Figure 1H). Both the novelty preference index and discrimination index were markedly reduced in SDN compared with CON (novelty preference index: 0.35 ± 0.16 vs. 0.77 ± 0.12, *p* < 0.0001; discrimination index: −0.29 ± 0.31 vs. 0.55 ± 0.23, *p* < 0.0001; Figure 1G,H). SDP significantly improved both measures relative to SDN and SDS (novelty preference index: 0.61 ± 0.10 vs. 0.35 ± 0.16 and 0.35 ± 0.14, *p* = 0.0004 and 0.0003, respectively; discrimination index: 0.23 ± 0.20 vs. −0.29 ± 0.31 and −0.30 ± 0.28, *p* = 0.0004 and 0.0003, respectively), although both indices remained modestly lower than CON (*p* = 0.042 for both; Figure 1G,H).

In the 24‐h NOR test, one‐way ANOVA also showed significant group effects on the novelty preference index (F (3, 36) = 16.97, *p* < 0.0001; Figure 1I) and discrimination index (F (3, 36) = 16.85, *p* < 0.0001; Figure 1J). SDN displayed significantly lower novelty preference and discrimination indices than CON (novelty preference index: 0.31 ± 0.27 vs. 0.87 ± 0.17, *p* < 0.0001; discrimination index: −0.38 ± 0.54 vs. 0.73 ± 0.33, *p* < 0.0001; Figure 1I,J), whereas SDP restored both measures to levels not significantly different from CON (novelty preference index: 0.81 ± 0.07 vs. 0.87 ± 0.17, *p* = 0.939; discrimination index: 0.63 ± 0.13 vs. 0.73 ± 0.33, *p* = 0.944). In contrast, SDP remained significantly higher than both SDN and SDS (novelty preference index: *p* < 0.0001 and 0.004, respectively; discrimination index: *p* < 0.0001 and 0.004, respectively), indicating improved short‐term and longer‐term recognition performance.

Together, propofol‐treated rats showed behavioral performance closer to control levels than SDN and SDS.

### 3.2 Propofol Increases SO Density During Recovery Sleep

EEG/EMG were recorded in CON, SDS, and SDP groups. Because the SDN group did not undergo recovery sleep, no EEG/EMG were collected for that group (Figure 2A).

Grand‐average SO and spindle waveforms are shown in Figure 2B,G for visualization. Importantly, SO density was significantly higher in SDP than in SDS (11.13 ± 0.14 vs. 9.49 ± 1.26 events/min, *p* = 0.040; Figure 2C), whereas SO amplitude, frequency, and duration did not differ consistently between groups (Figure 2D–F). Quantitative spindle metrics, including density, amplitude, frequency, and duration, likewise showed no significant group differences (Figure 2H–K).

These findings indicate that propofol primarily increased SO occurrence during recovery sleep, whereas other SO and spindle metrics remained largely unchanged across groups.

### 3.3 Propofol Treatment Realigns SO–Spindle Coupling to the SO Up‐State

**FIGURE 3:**
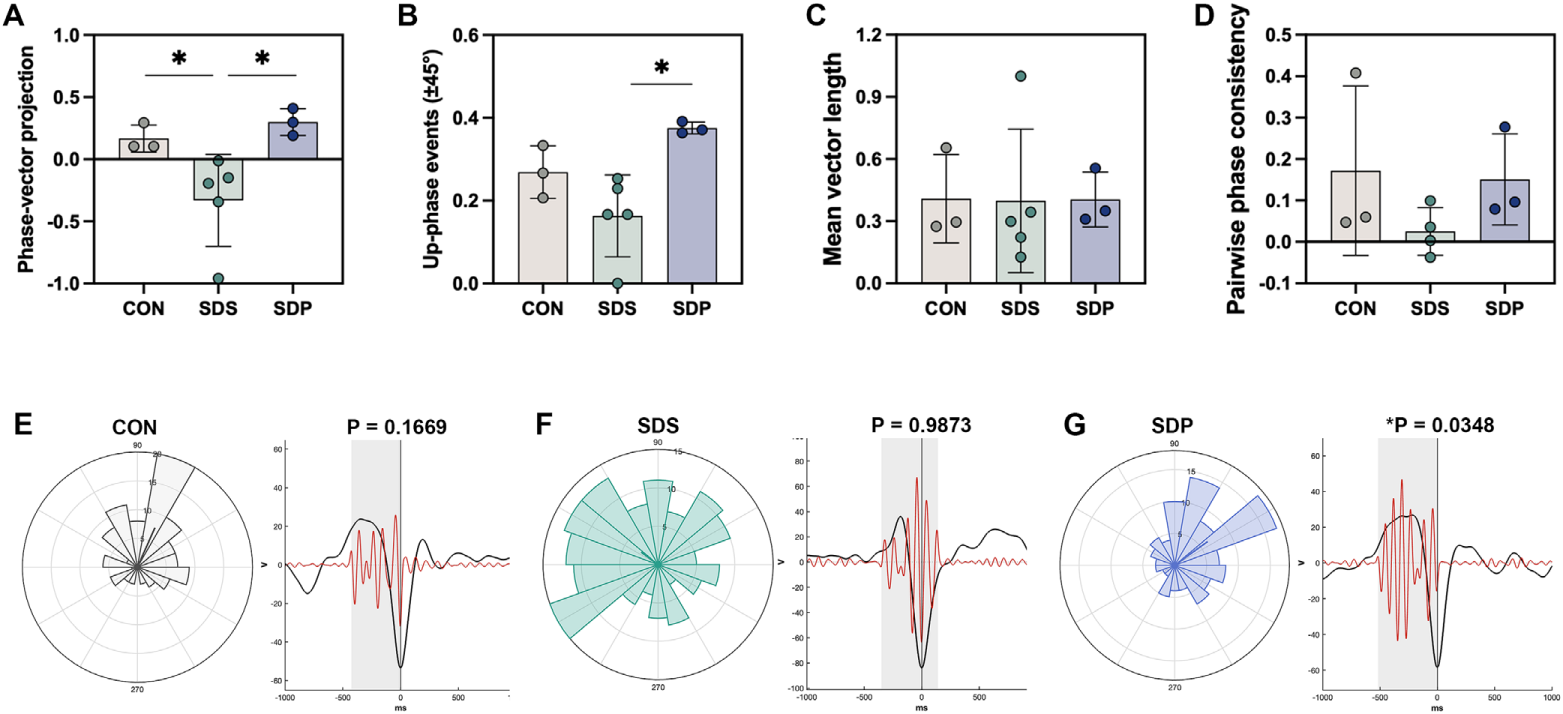
SO–spindle coupling during SWS. (A) Phase‐vector projection relative to the SO shows reduced alignment after SD with natural recovery (SDS) and enhanced alignment after propofol‐assisted recovery (SDP) relative to SDS. (B) Proportion of up‐phase spindles (±45° around the SO up‐phase) is increased in SDP versus SDS. (C) Mean vector length (MVL) summarizes coupling strength; (D) pairwise phase consistency across events/animals. (E–G) Circular histograms of spindle phase at SO timing with corresponding SO‐locked waveforms; Rayleigh/*V*‐test *p* values shown (significant phase preference in SDP, not in CON or SDS). Data are shown as mean ± SD with individual animals overlaid; *n* = 3–5 rats per group. Group comparisons for panels A–D were performed using Kruskal–Wallis tests, followed by Dunn's multiple‐comparisons test where appropriate. Circular analyses for panels E–G used *V*‐tests. The *p* values refer to the differences of variables between the groups, *p < 0.05.

Coupling analyses revealed that SD disrupted the preferred timing of spindle onsets relative to the SO cycle. Specifically, Vproj shifted from positive values in CON to negative values in SDS (0.17 ± 0.11 vs. −0.33 ± 0.37), and this displacement was significantly reversed in SDP (0.30 ± 0.11; CON vs. SDS, *p* = 0.036; SDP vs. SDS, *p* = 0.036; Figure 3A). Likewise, the proportion of up‐phase events was higher in SDP than in SDS (0.38 ± 0.01 vs. 0.16 ± 0.10, *p* = 0.036; Figure 3B), indicating a shift of spindle timing back toward the SO up‐state. In contrast, coupling strength and across‐event phase consistency showed no significant group differences. *V*‐tests further showed a significant up‐state preference only in SDP (CON *p* = 0.167, SDS *p* = 0.987, SDP *p* = 0.035; Figure 3E–G).

Thus, these findings demonstrate that propofol‐treated rats exhibited spindle timing that more consistently aligned with the SO up‐state.

### 3.4 Propofol Reverses the SD‐Induced NKCC1/KCC2 Imbalance

**FIGURE 4:**
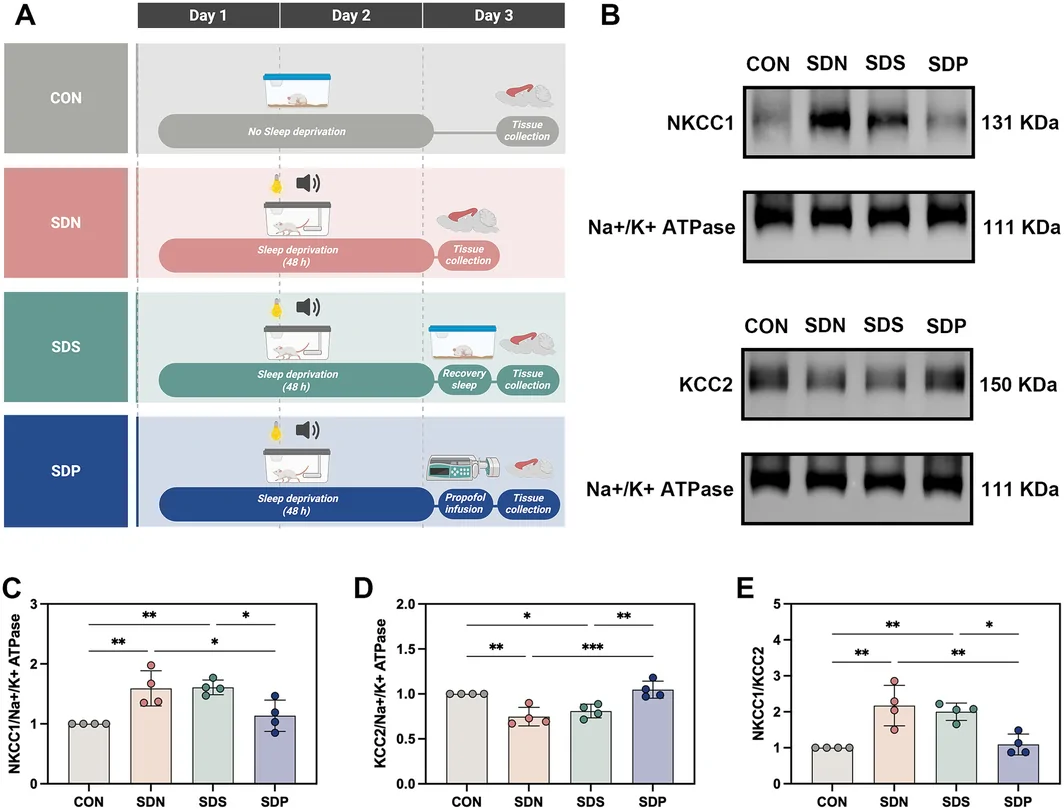
Cortical chloride cotransporters after SD and recovery. (A) Sampling windows. (B) Representative immunoblots of cortical NKCC1 and KCC2 with Na^+^/K^+^‐ATPase as the loading control. (C–E) Quantification in cortex: (C) NKCC1 normalized to Na^+^/K^+^‐ATPase, (D) KCC2 normalized to Na^+^/K^+^‐ATPase, and (E) NKCC1/KCC2 ratio. SDN showed higher NKCC1 and lower KCC2 compared with CON, resulting in an increased NKCC1/KCC2 ratio. SDS and SDP groups showed intermediate values, with SDP numerically closer to CON. Data are shown as mean ± SD with individual animals overlaid; *n* = 4 rats per group. One‐way ANOVA across groups with Tukey post hoc for NKCC1, KCC2, and the NKCC1/KCC2 ratio. The *p* values refer to the differences of variables between the groups, *p < 0.05, **p < 0.01, ***p < 0.001.

To explore the mechanism underlying propofol's restoration of SO–spindle coupling, we examined whether propofol corrected SD‐induced dysregulation of chloride cotransporters. Frontal and parietal cortices were collected immediately after SD completion in SDN, after spontaneous recovery in SDS, and after propofol‐supported recovery in SDP; CON tissue was obtained at time‐matched points for comparison (Figure 4A). One‐way ANOVA showed significant group effects on NKCC1 expression (F (3, 12) = 9.218, *p* = 0.0019), KCC2 expression (F (3, 12) = 13.12, *p* = 0.0004), and the NKCC1/KCC2 ratio (F (3, 12) = 12.91, *p* = 0.0005). Western blot analyses showed that SDN rats exhibited increased NKCC1 expression and reduced KCC2 expression relative to CON (NKCC1: 1.59 ± 0.29 vs. 1.00 ± 0.00, *p* = 0.007; KCC2: 0.75 ± 0.10 vs. 1.00 ± 0.00, *p* = 0.004), consistent with an elevated NKCC1/KCC2 ratio (2.17 ± 0.56 vs. 1.00 ± 0.00, *p* = 0.002; Figure 4B–E). Propofol reversed these changes: compared with SDN and SDS, SDP showed lower NKCC1 expression (1.14 ± 0.26 vs. 1.59 ± 0.29, *p* = 0.037; 1.14 ± 0.26 vs. 1.61 ± 0.12, *p* = 0.030), higher KCC2 expression (1.05 ± 0.10 vs. 0.75 ± 0.10, *p* = 0.001; 1.05 ± 0.10 vs. 0.81 ± 0.08, *p* = 0.006), and a reduced NKCC1/KCC2 ratio (1.09 ± 0.29 vs. 2.17 ± 0.56, *p* = 0.003; 1.09 ± 0.29 vs. 2.00 ± 0.24, *p* = 0.012), with no significant difference from CON in any of these measures (Figure 4B–E). These molecular changes were observed alongside the electrophysiological differences in SO occurrence and coupling timing.

Together, these results show that propofol reversed SD‐induced changes in cortical chloride cotransporters.

## 4 Discussion

This study demonstrates that propofol administered during recovery after SD restores spatial and recognition memory, with retention maintained at 24 h. On sleep EEG, propofol increased the expression of slow oscillations and markedly improved the phase‐accurate alignment of spindle onsets to the SO up‐state. At the molecular level, propofol reversed the NKCC1/KCC2 imbalance induced by SD, supporting a chloride homeostasis mechanism consistent with enhanced coupling precision.

Sleep loss is strongly associated with cognitive decline across clinical and experimental settings. In humans, even a single night of SD reduces hippocampal activity during episodic encoding and yields poorer subsequent recall [33]. Meta‐analytic data in healthy adults show that acute SD and short‐term sleep restriction produce measurable neurocognitive deficits, with attention and working memory especially vulnerable [34, 35]. Rodent work similarly shows impaired memory consolidation after brief deprivation [36]. Consistent with this literature, our study observed robust behavioral deficits after deprivation and recovery of spatial and recognition memory with propofol‐supported recovery sleep, aligning post‐deprivation behavioral improvement with established sleep–memory relationships.

Pharmacologically, propofol promotes slow‐delta activity and coherent frontal rhythms consistent with NREM‐like organization [12]. In the present study, the dose of 20 mg·kg^−1^·h^−1^ was selected for three reasons. First, our model was designed to reflect the clinical context in which propofol is titrated for ICU sedation rather than used for general anesthesia [37]. Second, previous rat studies have used 10, 20, and 40 mg·kg^−1^·h^−1^ as a dose range for propofol sedation, with 20 mg·kg^−1^·h^−1^ corresponding to conscious or light sedation and 40 mg·kg^−1^·h^−1^ producing a deeper, general anesthesia‐like state [38]. Third, our previous work supported 20 mg·kg^−1^·h^−1^ as a sub‐anesthetic neuroprotective dose: 40 mg·kg^−1^·h^−1^ was considered a maintenance dose for general anesthesia in rats, whereas 20 mg·kg^−1^·h^−1^ showed neuroprotective effects, and higher doses such as 35, 36, or 72 mg·kg^−1^·h^−1^ failed to provide benefit or even worsened neurological outcomes [39]. Therefore, 20 mg·kg^−1^·h^−1^ was used here as a sub‐anesthetic, sedation‐like intervention to support post‐deprivation recovery sleep. Importantly, SO–spindle coupling was analyzed during recovery sleep after the propofol intervention, not during the infusion itself, reducing the likelihood that the observed coupling reflected burst‐suppression activity.

SOs and sleep spindles provide complementary temporal windows for off‐line plasticity during NREM sleep, with SOs organizing cortical up–down states and spindles reflecting thalamocortical activity [18]. In our data, propofol increased SO density and normalized SO waveform shape during recovery, whereas canonical spindle amplitude, density, frequency, and duration changed little. This dissociation is plausible because SD and subsequent recovery primarily modulate cortical homeostatic pressure expressed in SOs [40], whereas spindle generators are constrained by reticular‐thalamic circuitry and state‐dependent neuromodulation, making spindle metrics less sensitive to short‐window pharmacologic support [32]. These observations identify a propofol‐dependent increase in SO availability during recovery sleep and motivate the subsequent analysis of phase‐specific coupling.

Coordinated coupling among sleep rhythms provides brief windows for long‐range information transfer during systems consolidation. SOs organize cortical up‐states that gate thalamocortical spindles, and the phase‐accurate alignment of spindles to the SO cycle robustly predicts next‐day memory in humans [18, 20]. Mechanistic studies of SO–spindle–ripple coupling suggest that SO up‐states provide an excitability window, within which spindles can gradually increase neuronal firing rates and facilitate hippocampal ripple‐associated replay. Beyond ripple facilitation, spindle‐band synchronization may also open and maintain channels for cross‐regional communication, allowing reactivated hippocampal information to be transmitted to distributed cortical networks and progressively stabilized during systems consolidation [41, 42]. Our data show that recovery sleep with propofol increases SO availability and sharpens the temporal preference of spindle onsets for the SO up‐state, while overall spindle counts and amplitudes remain relatively stable. These findings suggest that propofol may restore the top‐down neocortical–thalamocortical timing scaffold. Thus, although hippocampal ripples were not directly recorded, the restored SO up‐state alignment in SDP rats may create favorable timing conditions for hippocampal replay and hippocampal‐dependent spatial memory consolidation during recovery sleep.

At the molecular level, the precision of SO–spindle coupling depends on the inhibitory environment that shapes cortical up–down dynamics. Chloride homeostasis determines the polarity of GABA_A_ currents: KCC2 extrudes Cl^−^ while NKCC1 imports it, so a lower NKCC1/KCC2 ratio drives a more negative *E*_*Cl*_ strengthens hyperpolarizing GABA, slows firing, and promotes population synchrony [43, 44, 45]. Such enhanced inhibition stabilizes the SO framework generated by recurrent cortical networks, sharpening down‐to‐up transitions and supporting more robust up‐states [46, 47]. In this context, spindles are initiated by thalamic generators but are gated by cortical state, so more stable up‐states favor phase‐accurate spindle initiation [32]. Propofol potentiates GABA_A_ signaling and promotes neuronal hyperpolarization [48], consistent with improved inhibitory tone during recovery sleep. In our study, propofol decreased the cortical NKCC1/KCC2 ratio, accompanied by higher SO density and sharper spindle alignment to the SO up‐state, indicating that chloride‐transport‐dependent strengthening of inhibition can support the electrophysiological conditions necessary for consolidation‐relevant coupling.

Several limitations should be noted. We did not include hippocampal recordings, so the relationship between cortical SO–spindle dynamics and deeper network events such as ripples remains to be clarified. The EEG/EMG cohort was relatively small and independent, which may limit statistical power for detecting subtle between‐group differences in some EEG‐derived measures; nevertheless, the nonparametric sensitivity analyses summarized in Table S1 supported the interpretation of the main results. The use of adult male rats in an acute 48‐h sleep‐deprivation model may limit generalizability to broader biological and clinical contexts. Our mechanistic focus was on chloride homeostasis; other circuit‐ and neuromodulatory factors may also influence SO–spindle timing and warrant future study. These considerations outline avenues for follow‐up work but do not affect the central observation that propofol sharpened up‐state SO–spindle alignment during recovery sleep.

## 5 Conclusion

This study demonstrates that propofol administered during recovery from SD improves cognition by increasing SO density and restoring phase‐accurate alignment of spindle onsets to the SO up‐state. The resulting up‐state–precise SO–spindle coupling represents a sleep‐EEG phenotype associated with recovery of spatial performance on the BM and with improved short‐term and longer‐term recognition memory on NOR. At the molecular level, the reduced cortical NKCC1/KCC2 ratio suggests a shift toward a more hyperpolarizing chloride equilibrium potential, which may synergize with propofol‐mediated GABA_A_ receptor potentiation to strengthen inhibitory tone, stabilize SO down‐to‐up state transitions, and support more precise SO–spindle coupling. Together, these findings identify SO–spindle alignment as a potential biomarker of post‐deprivation cognitive vulnerability and highlight chloride‐transport mechanisms as tractable targets for recovery‐sleep‐oriented interventions.

## Author Contributions

Study concept and design: Chenyi Yang and Haiyun Wang. Experimental work: Huan Liu, Lin Zhang, and Xinyi Wang. Acquisition, analysis, or interpretation of data: Chenyi Yang. Drafting of the manuscript: Chenyi Yang. Critical revision of the manuscript for important intellectual content: All authors. Statistical analysis: Chenyi Yang. Funding acquisition: Chenyi Yang and Haiyun Wang. Administrative, technical, or material support: Chenyi Yang, Xinyi Wang, and Haiyun Wang. Study supervision: Haiyun Wang.

## Funding

This work was sponsored by the Tianjin Health Research Project (Grant No. TJWJ2023QN042), Tianjin Outstanding Young Health Professionals Development Program (Grant No. YQ2026067), and State Key Laboratory of Advanced Medical Materials and Devices Research Grant (Grant No. YGSKL‐TJU‐2025‐KF22).

## Ethics Statement

All animal procedures were approved by the Animal Ethical and Welfare Committee of Tianjin University (Approval No. TJUE‐2025‐247). All experiments were conducted in accordance with institutional guidelines, and every effort was made to minimize animal suffering.

## Conflicts of Interest

The authors declare no conflicts of interest.

## Supporting information


**Table S1:** Statistical diagnostics and non‐parametric sensitivity analyses for principal outcomes.

## Data Availability

The data that support the findings of this study are available from the corresponding author upon reasonable request.
